# Retrospective clinical study of endoscopic transfrontal approach vs. transSylvian-transinsular craniotomy for hypertensive intracerebral hemorrhage in basal ganglia: efficacy comparison and value of anatomical cognition of Sylvian fissure

**DOI:** 10.3389/fsurg.2026.1860820

**Published:** 2026-06-18

**Authors:** Wen-Song Su, Xiao-Qiong Su, Xu-Xiang Yu, Feng-Lin Zhang, Xin-Hua Tian, Zhong Liu

**Affiliations:** 1 Department of Central Intensive Care Unit, School of Medicine, Zhongshan Hospital of Xiamen University, Xiamen University, Xiamen, China; 2 College of Plant Science and Technology, Hunan Biological and Electromechanical Polytechnic, Changsha, China; 3 Department of Neurosurgery, School of Medicine, Zhongshan Hospital of Xiamen University, Xiamen University, Xiamen, Fujian, China

**Keywords:** anatomical cognition, basal ganglia, hypertensive intracerebral hemorrhage, neuroendoscopy, sylvian fissure

## Abstract

**Background:**

To compare the efficacy of endoscopic transfrontal and transSylvian-transinsular hematoma evacuation for basal ganglia hypertensive intracerebral hemorrhage (HICH), identify independent prognostic factors, and explore the value of the latter in improving young doctors’ anatomical cognition of the Sylvian fissure.

**Methods:**

A retrospective analysis was performed on 230 basal ganglia HICH patients, divided into the endoscopic group (*n* = 150) and craniotomy group (*n* = 80). Surgical efficacy, postoperative outcomes, prognostic factors, and Sylvian fissure-related anatomical cognition scores of 8 young doctors in the craniotomy group were analyzed.

**Results:**

The two groups had no significant differences in hematoma clearance rate (89.2 ± 7.5% vs. 87.6 ± 8.2%), postoperative rebleeding rate (4.0% vs. 5.0%) and 3-month favorable prognosis rate (62.0% vs. 58.8%) (all *P* > 0.05). The endoscopic group had shorter operation time, less intraoperative blood loss and lower complication rate (all *P* < 0.05). Preoperative GCS score, hematoma volume, intraoperative blood loss and postoperative pulmonary infection were indicated as independent prognostic factors. Young doctors’ Sylvian fissure anatomical theory and operative scores were significantly higher postoperatively (89.5 ± 6.2, 86.8 ± 7.5) than preoperatively (65.3 ± 8.7, 62.5 ± 9.3) (all *P* < 0.001).

**Conclusion:**

Both approaches have comparable core efficacy for basal ganglia HICH, with endoscopic surgery having minimally invasive advantages. As an exploratory observational analysis, this study suggests that transSylvian-transinsular craniotomy may help improve young doctors’ anatomical cognition of the Sylvian fissure and appears to offer certain clinical teaching value. Clinical selection of surgical methods should be based on the patient's specific condition, operator experience and hospital equipment conditions.

## 1 Introduction

Hypertensive intracerebral hemorrhage (HICH) is a devastating cerebrovascular disorder with high global incidence, disability, and mortality rates, imposing a substantial burden on healthcare systems worldwide (1–3). Epidemiological studies and systematic reviews suggest that HICH accounts for a significant proportion of cerebrovascular diseases, with basal ganglia hemorrhage being the most common subtype—often leading to severe neurological deficits due to compression of adjacent critical structures such as the internal capsule and basal ganglia nuclei (1, 4, 5). Timely and effective hematoma evacuation is considered as a core therapeutic strategy to improve patient outcomes, as indicated in international clinical guidelines (2, 6).

Currently, various surgical approaches are available for the treatment of basal ganglia HICH, including endoscopic surgery and traditional craniotomy. Endoscopic transfrontal surgery, as a minimally invasive technique, has gained increasing attention in recent years for its potential to reduce surgical trauma, with comparative studies and meta-analyses indicate its potential advantages in shortening operation time, reducing intraoperative blood loss, and lowering postoperative complication rates (7–9). In contrast, trans Sylvian-trans insular craniotomy leverages the natural anatomical space of the Sylvian fissure to access deep-seated hematomas (10, 11). This approach not only provides effective hematoma evacuation but also may provide unique opportunities for young neurosurgeons to enhance their understanding of complex cerebral anatomy, as the Sylvian fissure and insular region are regarded as key and difficult points in neurosurgical training (12, 13). However, the comparative efficacy of these two approaches in the treatment of basal ganglia HICH remains incompletely understood, and the key prognostic factors influencing long-term outcomes warrant further clinical investigation.

Previous studies have suggested several potential prognostic factors for HICH, including preoperative neurological status, hematoma volume, intraoperative parameters, and postoperative complications (2, 6, 14). Meanwhile, the choice of surgical approach is often tailored to patient-specific characteristics such as hematoma location, baseline condition, and surgical team expertise (15, 16). Despite the availability of multiple surgical techniques, there is a lack of targeted analysis focusing on the comparative value of endoscopic transfrontal surgery and transSylvian-transinsular craniotomy, as well as the integration of prognostic factor identification and clinical teaching value.

A major highlight and innovation of the present work is that it is the first study to conduct an exploratory observational analysis to investigate and quantify the potential educational value of transSylvian-transinsular craniotomy in improving young doctors’ anatomical cognition of the Sylvian fissure. Therefore, this study retrospectively analyzed 230 patients with basal ganglia HICH treated by the two aforementioned surgical methods. The objectives were to compare the clinical efficacy of the two approaches, identify independent baseline and surgical-related prognostic factors for 3-month favorable prognosis, and explore the clinical teaching value of transSylvian-transinsular craniotomy in improving young doctors’ anatomical cognition of the Sylvian fissure. The findings aim to provide evidence-based references for personalized surgical decision-making, prognostic evaluation, and neurosurgical education in the management of basal ganglia HICH.

## 2 Materials and methods

### 2.1 General information

A total of 246 adult patients were initially screened according to the inclusion and exclusion criteria between January 2019 and January 2024. Sixteen patients were excluded for the following reasons: seven did not meet the diagnostic criteria for basal ganglia HICH, five had severe comorbidities with surgical contraindications, and four had incomplete clinical data. Ultimately, 230 eligible patients were enrolled in this retrospective observational study.

The endoscopic group (EG) consisted of 150 consecutive patients with basal ganglia HICH. These patients were matched with a historical control group (craniotomy group, CG), which included 80 consecutive patients who underwent transSylvian-transinsular craniotomy during the same period at the Department of Neurosurgery, Zhongshan Hospital of Xiamen University.

**Figure 1:**
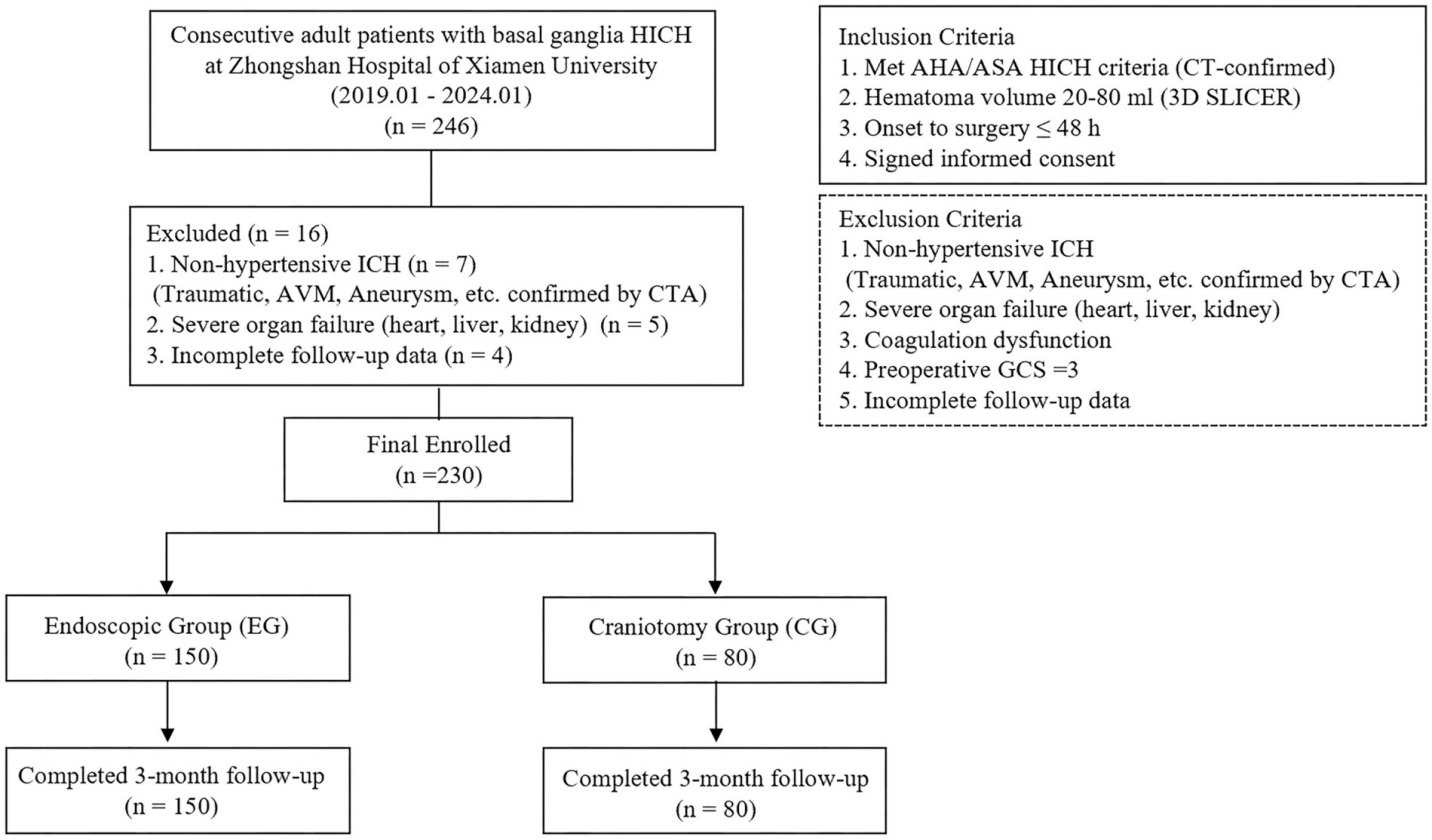
CONSORT-style participant flow diagram showing patient screening, enrollment, group allocation and follow-up in this retrospective study.

Preoperative and postoperative supportive treatments, including standardized blood pressure management, were consistent across all participants. All enrolled patients completed the 3-month postoperative follow-up, as illustrated in the CONSORT-style participant flow diagram (Figure 1).

All procedures performed in this study were approved by the Scientific Research Subcommittee of the Medical Ethics Committee of Zhongshan Hospital of Xiamen University, and informed consent was obtained from each patient or their family members.

#### 2.1.1 Inclusion criteria

(1) Conformed to the diagnostic criteria of HICH in the *Guidelines for the Management of Spontaneous Intracerebral Hemorrhage* issued by the American Heart Association/American Stroke Association (AHA/ASA) (6), and basal ganglia hemorrhage was confirmed by cranial Computed Tomography (CT); (2) Hematoma volume was 20∼80 mL (accurately calculated by open-source software 3D SLICER); (3) Time from onset to surgery ≤48 h; (4) Patients or their families signed the informed consent for surgery.

#### 2.1.2 Exclusion criteria

(1) Traumatic intracerebral hemorrhage, intracerebral hemorrhage due to vascular malformations or ruptured aneurysms [confirmed by computed tomography angiography (CTA)], and other non-hypertensive intracerebral hemorrhages; (2) Complicated with severe heart, liver, kidney and other organ failure; (3) Coagulation dysfunction; (4) Preoperative Glasgow Coma Scale (GCS) score = 3 points with poor expected prognosis; (5) Incomplete follow-up data.

### 2.2 Surgical methods

All surgeries were performed by senior attending neurosurgeons with more than 10 years of clinical experience (17, 18), and 8 young doctors with ≤5 years of clinical experience participated in the craniotomy group as surgical assistants.

#### 2.2.1 Craniotomy group (hematoma evacuation via transSylvian-transinsular approach)

**Figure 2:**
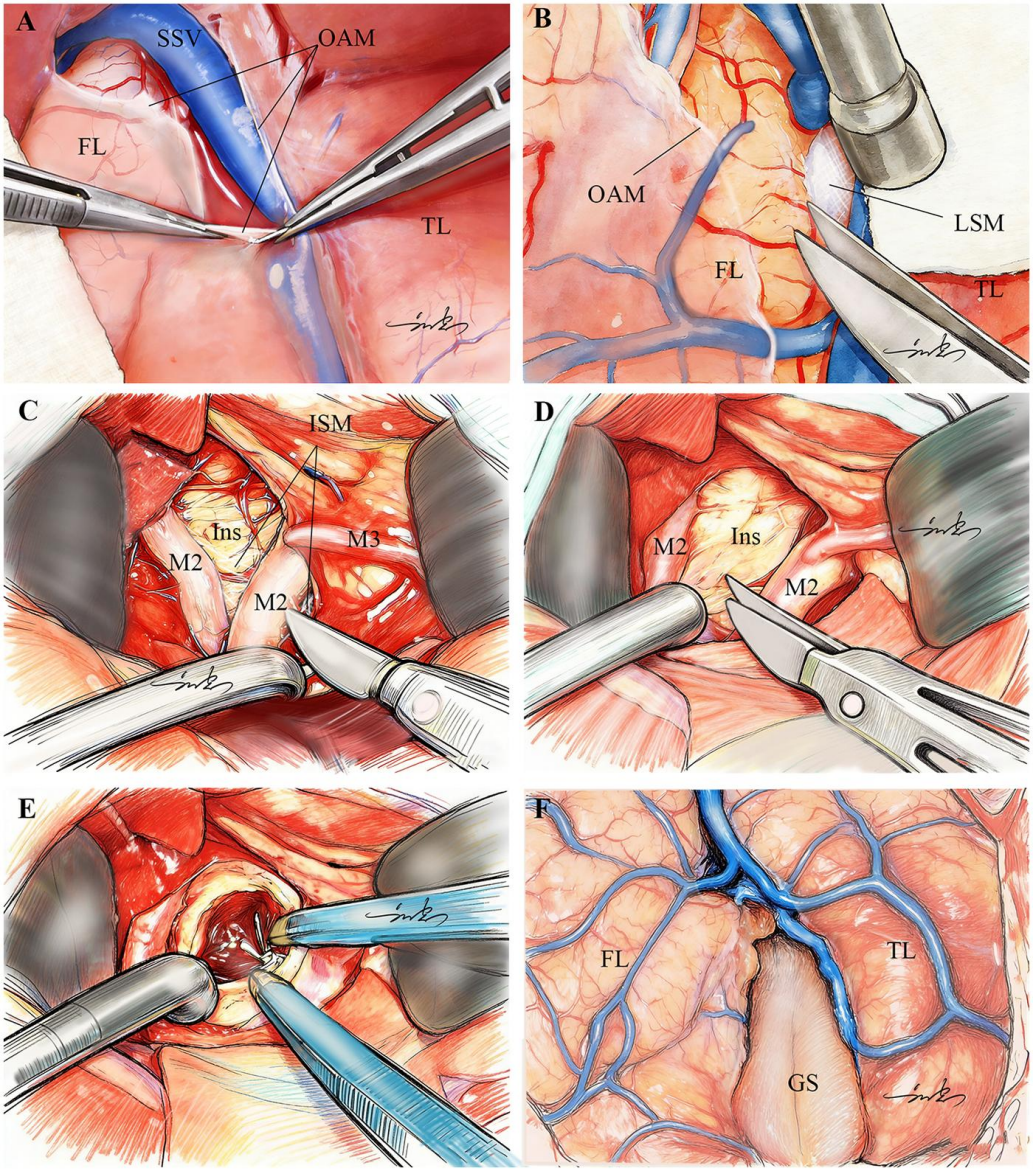
Hand-drawn diagram showing the transSylvian-transinsular evacuation of right basal ganglia hematoma and the membranous structure of the sylvian fissure. **A**, Using two forceps to separate the outer arachnoid membrane (OAM) to expose and protect the underlying superficial Sylvian vein (SSV); **B**, Separating the lateral Sylvian membrane (LSM), including the arachnoid trabeculae between veins, between veins and cerebral pia mater, or between the pia mater of frontal and temporal brain tissue; **C**, Separating the intermediate Sylvian membrane (ISM), including the arachnoid trabeculae between arteries, between arteries and brain tissue, or between frontal and temporal brain tissue; the M2 segment of the middle cerebral artery (MCA) on the insular surface is visible. **D**, Sharply incising the pia mater on the surface of the insula (Ins), and the deep part is the basal ganglia hematoma; **E**, Electrocoagulating the surface of the insula to expose and evacuate the hematoma; **F**, After hematoma evacuation, the brain tissue pressure decreases, the tension of the superficial Sylvian vein decreases, and the main trunk is well protected. FL, frontal lobe; TL, temporal lobe; GS, gelatin sponge.

**Figure 3:**
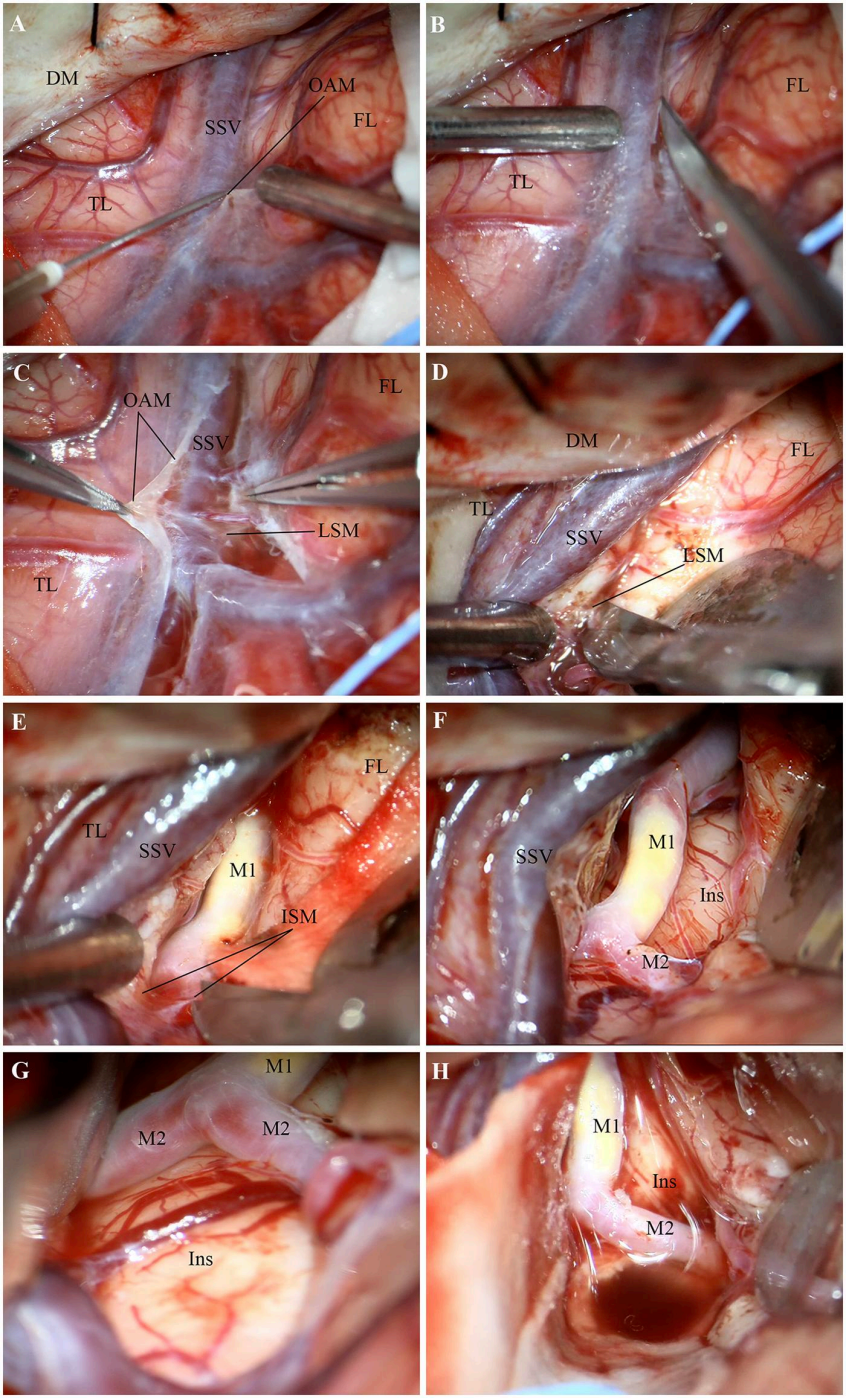
Intraoperative diagram showing the transSylvian-transinsular evacuation of basal ganglia hematoma and the membranous structure of the sylvian fissure. **A**, Using a 1 mL syringe needle to make a small incision in the loose outer arachnoid membrane (OAM) in the middle segment of the Sylvian fissure; **B,C**, Using sharp (**B**) combined with blunt separation with two forceps (**C**) to separate the OAM to expose and protect the underlying superficial Sylvian vein (SSV); **D**, Separating the arachnoid trabeculae between veins and brain tissue, i.e., the lateral Sylvian membrane (LSM), to expose the underlying middle cerebral artery (MCA); **E**, Separating the intermediate Sylvian membrane (ISM), including the arachnoid trabeculae between arteries, between arteries and brain tissue, or between frontal and temporal brain tissue; **F,G**, Exposing the surface of the insula (Ins); **H**, After hematoma evacuation, the brain tissue pressure decreases. FL, frontal lobe; TL, temporal lobe; DM, dura mater.

**Figure 4:**
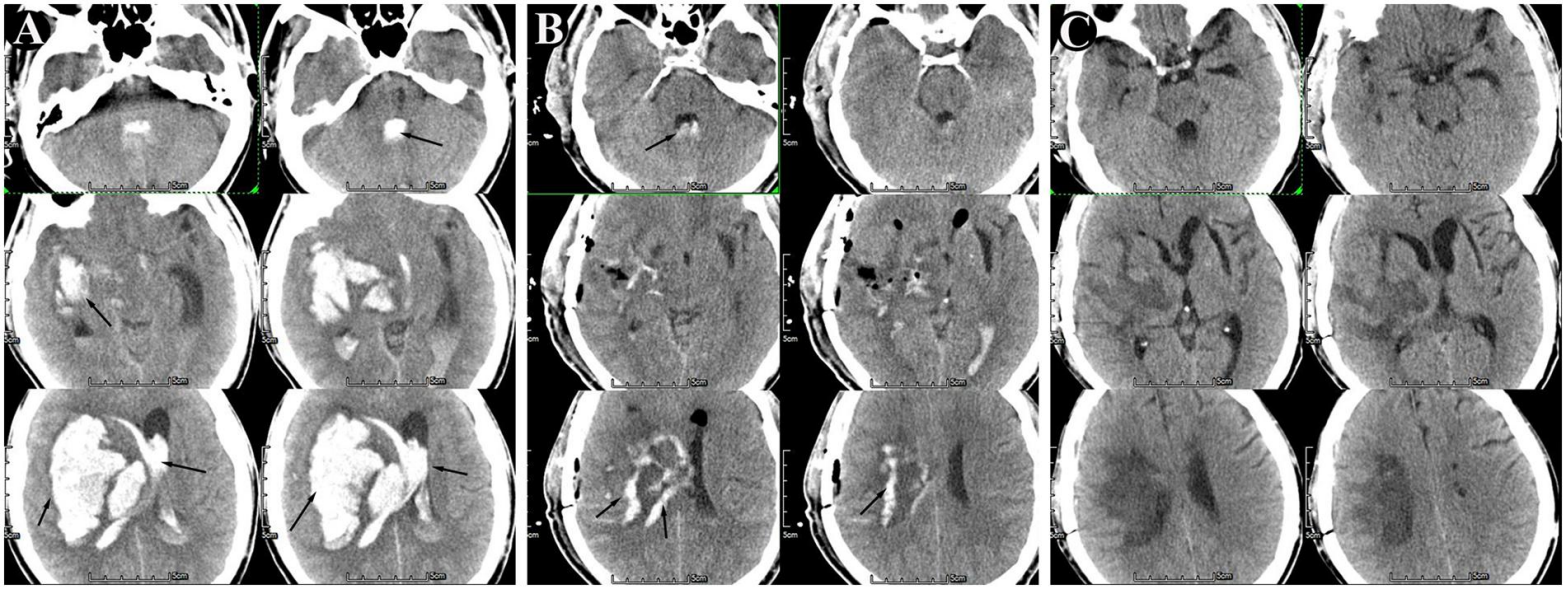
Illustrative case of a 48-year-old male with right basal ganglia HICH treated by transSylvian-transinsular craniotomy. **A**, Preoperative axial cranial CT showing right basal ganglia hemorrhage rupturing into the lateral ventricle, with diffuse and irregular hematoma morphology; **B**, Postoperative 24-hour CT demonstrating satisfactory hematoma evacuation; **C**, Postoperative 2-week CT showing stable intracranial condition with no rebleeding or obvious cerebral edema. Black arrows indicate the location of hematoma (**A**) or postoperative residual hematoma (**B**). HICH, hypertensive intracerebral hemorrhage; CT, computed tomography.

Under general anesthesia, patients were placed in the supine position with the head appropriately rotated contralaterally. A standard frontotemporal curved incision and bone flap craniotomy were performed, followed by dural incision for surgical exposure. Under microscopic visualization, the Sylvian fissure was dissected along its natural anatomical plane with careful protection of the superficial Sylvian vein and middle cerebral artery branches. After exposing the insular surface, a small cortical incision was made at the avascular insular region to access the hematoma cavity. Microscopic hematoma aspiration and meticulous hemostasis were performed, followed by layered craniotomy closure. Key intraoperative procedures are illustrated in Figures 2–4.

#### 2.2.2 Endoscopic group (neuroendoscopic hematoma evacuation via transfrontal approach)

**Figure 5:**
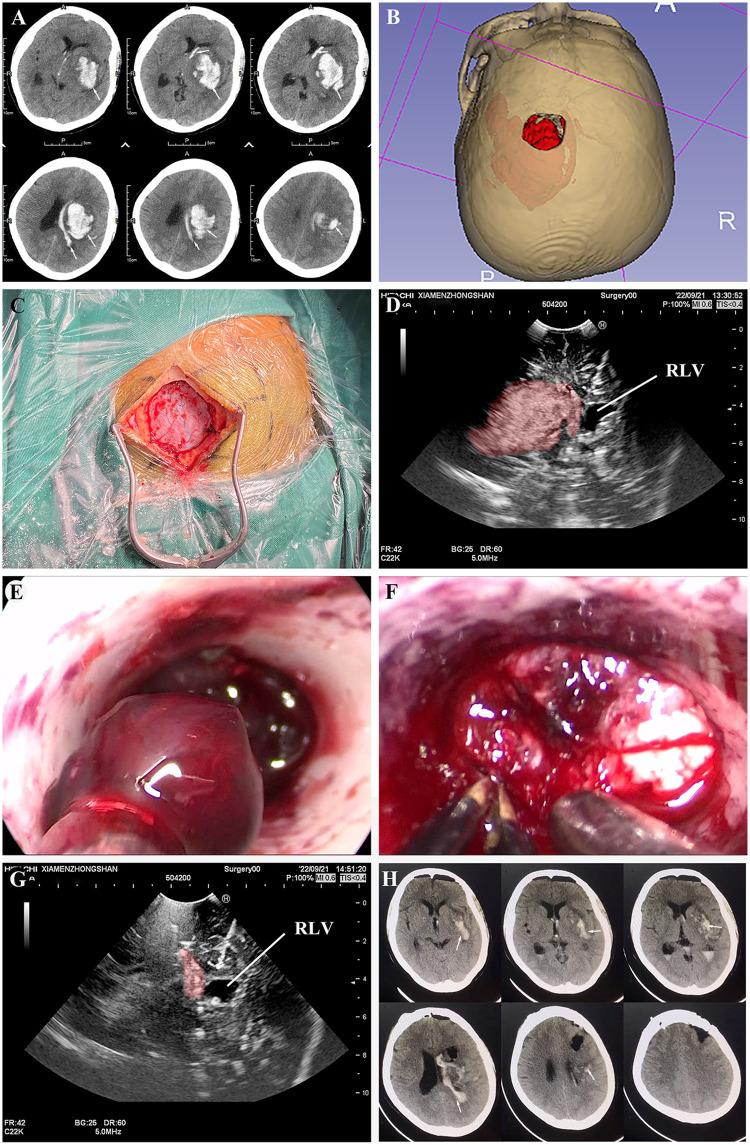
Illustrative case of a 56-year-old female with left basal ganglia HICH treated by endoscopic transfrontal surgery. **A**, Preoperative axial cranial CT showing left basal ganglia hemorrhage rupturing into the lateral ventricle; **B**, 3D Slicer reconstruction of the hematoma with planned bone window position and size; **C**, Intraoperative view of the left frontal bone window; **D**, Intraoperative coronal ultrasound demonstrating the left basal ganglia hematoma (red shadow) rupturing into the lateral ventricle; **E**, Endoscopic view during hematoma evacuation; **F**, Surgical field after complete hematoma clearance; **G**, Post-evacuation ultrasound showing near-complete removal of the basal ganglia hematoma, with minimal residual intraventricular hematoma (red shadow); **H**, Postoperative axial cranial CT confirming hematoma clearance consistent with intraoperative ultrasound findings. White arrows indicate the location of hematoma or postoperative residual hematoma. HICH, hypertensive intracerebral hemorrhage; CT, Computed Tomography; RLV, right lateral ventricle.

Under general anesthesia in the supine position, accurate preoperative localization of the hematoma was performed based on cranial CT images to avoid functional cortical areas. A small frontal craniotomy was created, and a transfrontal working channel was established using a neuroendoscope. Hematoma was aspirated under endoscopic high-definition visualization, with targeted bipolar coagulation for active bleeding. After adequate hematoma evacuation and hemostasis, routine layered cranial closure was completed. Representative intraoperative findings are shown in Figure 5.

### 2.3 Observation indicators

#### 2.3.1 Baseline characteristics

Demographic data [gender, age, body mass index (BMI)], preoperative clinical indicators [preoperative systolic blood pressure [SBP], diastolic blood pressure [DBP], admission GCS score, hematoma volume [HV]], and baseline blood lipid indicators (total cholesterol [TC], triglyceride [TG], low-density lipoprotein cholesterol [LDL-C], high-density lipoprotein cholesterol [HDL-C]), comorbidities (diabetes mellitus, coronary heart disease).

#### 2.3.2 Surgery, postoperative outcome and prognostic indicators

(1) Surgery-related indicators: Operation time, intraoperative blood loss; (2) Hematoma evacuation indicators: Cranial CT was rechecked 24 h after surgery, and hematoma volume was calculated by 3D SLICER to obtain hematoma clearance rate [hematoma clearance rate = (preoperative hematoma volume - postoperative residual hematoma volume)/preoperative hematoma volume ×100%]; postoperative rebleeding rate within 7 days (residual hematoma volume increased by ≥30% or new bleeding foci appeared); (3) Postoperative complication indicators: Incidence of postoperative cerebral infarction, intracranial infection, pulmonary infection, lower extremity deep venous thrombosis (DVT) and total complication rate; (4) Prognostic indicator: 3-month mRS score, in which 0∼2 points were defined as favorable prognosis, 3∼6 points as unfavorable prognosis (7).

#### 2.3.3 Anatomical cognition of sylvian fissure assessment indicators

For the young doctors (8 in total) who participated in the craniotomy group, anatomical theory assessment (full score 100 points) and surgical operation assessment (focusing on Sylvian fissure anatomy identification and separation, full score 100 points) were used to evaluate their anatomical cognition level before surgery and 6 months after surgery.

##### 2.3.3.1 Anatomical theoretical assessment (full score 100 points)

A closed-book written test with a duration of 90 min, designed to assess young doctors’ mastery of Sylvian fissure anatomical theory and clinical application ability. The test consists of three parts: single-choice questions (10 questions, 5 points each, total 50 points), multiple-choice questions (4 questions, 5 points each, total 20 points), and essay questions (3 questions, 10 points each, total 30 points). The assessment covers 5 core dimensions: Sylvian fissure membranous structure classification, Sylvian fissure vascular distribution rules, anatomical relationship between Sylvian fissure and insula/basal ganglia, key points of Sylvian fissure separation, and complication prevention in Sylvian fissure surgery, with reference to the anatomical characteristics and surgical application requirements of the Sylvian fissure described in related literatures. The detailed test questions, answer key, and scoring standards are shown in Supplementary Material 1.

##### 2.3.3.2 Operative performance assessment (full score 100 points)

The assessment is based on intraoperative operation video review and on-site surgical observation, scored by a panel of 3 senior neurosurgery physicians (attending physician or above) with more than 10 years of clinical experience in transSylvian-transinsular craniotomy. The final score is the average score of the panel (excluding the highest and lowest scores if the score difference exceeds 10 points, and re-evaluated by the panel). The assessment covers 4 core dimensions, with detailed scoring criteria formulated with reference to the anatomical characteristics, surgical techniques, and complication prevention requirements of Sylvian fissure dissection described in related literatures. The detailed scoring standards are shown in Supplementary Material 2.

### 2.4 Statistical methods

Statistical analyses were performed using SPSS 26.0. Measurement data were presented as mean ± standard deviation. Comparisons between two groups were conducted with independent-samples t-test, and paired t-test was used for pre- and post-operative score comparisons in young doctors. Categorical data were expressed as percentages and compared using the *χ*^2^ test, with *χ*^2^ trend test for ordered categorical variables. Univariate analysis was first performed to screen prognostic factors (*P* < 0.10 for inclusion), followed by multivariate logistic regression to identify independent predictors of 3-month unfavorable prognosis. Dummy variables were set for ordered categorical variables (admission GCS score, preoperative hematoma volume) in regression models. Confounder adjustment was applied when analyzing surgical prognostic factors by incorporating independent baseline factors. A two-sided *P* < 0.05 was considered statistically significant.

## 3 Results

### 3.1 Comparison of baseline data between the two groups

A total of 230 patients with basal ganglia HICH were included in this study, including 150 cases in the EG and 80 cases in the CG. The baseline characteristics of the two groups were comparable, with no statistically significant differences in all indicators (all *P* > 0.05), supporting the reliability of subsequent efficacy comparisons.

In terms of demographic data, the EG included 92 males (61.3%) and 58 females (38.7%) with an average age of (61.8 ± 8.9) years and an average BMI of (24.3 ± 3.1) kg/m^2^, while the CG included 47 males (58.8%) and 33 females (41.2%) with an average age of (62.9 ± 8.5) years and an average BMI of (23.9 ± 2.8) kg/m^2^ (all *P* > 0.05). For preoperative clinical indicators, the average preoperative SBP (178.5 ± 22.3 vs. 180.2 ± 23.1 mmHg), DBP (102.6 ± 15.4 vs. 103.8 ± 14.9 mmHg), admission GCS score (10.3 ± 2.0 vs. 9.9 ± 2.4 points) and hematoma volume (44.9 ± 12.6 vs. 45.8 ± 12.1 mL) were similar between the two groups (all *P* > 0.05), indicating comparable disease severity at admission. Regarding baseline blood lipid indicators, the average TC (5.23 ± 1.05 vs. 5.31 ± 1.12 mmol/L), TG (1.86 ± 0.62 vs. 1.92 ± 0.58 mmol/L), LDL-C (3.35 ± 0.89 vs. 3.42 ± 0.95 mmol/L) and HDL-C (1.12 ± 0.25 vs. 1.08 ± 0.28 mmol/L) showed no significant differences between the two groups (all *P* > 0.05). In terms of comorbidities, the incidence of diabetes mellitus (21.3% vs. 18.8%) and coronary heart disease (18.7% vs. 17.5%) was balanced between the two groups (*P* = 0.643, *P* = 0.818). The specific baseline data are summarized in Table 1.

**Table 1:** Comparison of clinical baseline parameters between the Two groups.

Clinical Parameters	EG (*n* = 150)	CG (*n* = 80)	*P*
Demographic data			
Sex (male/female, *n*/%)	92/58 (61.3/38.7)	47/33 (58.8/41.2)	0.664
Age (years)	61.8 ± 8.9	62.9 ± 8.5	0.357
BMI (kg/m^2^)	24.3 ± 3.1	23.9 ± 2.8	0.340
Preoperative clinical indicators			
Preoperative SBP (mmHg)	178.5 ± 22.3	180.2 ± 23.1	0.588
Preoperative DBP (mmHg)	102.6 ± 15.4	103.8 ± 14.9	0.596
Admission GCS score (points)	10.3 ± 2.0	9.9 ± 2.4	0.177
Hematoma volume (mL)	44.9 ± 12.6	45.8 ± 12.1	0.609
Baseline blood lipid indicators			
TC (mmol/L)	5.23 ± 1.05	5.31 ± 1.12	0.591
TG (mmol/L)	1.86 ± 0.62	1.92 ± 0.58	0.484
LDL-C (mmol/L)	3.35 ± 0.89	3.42 ± 0.95	0.615
HDL-C (mmol/L)	1.12 ± 0.25	1.08 ± 0.28	0.294
Comorbidities (*n*, %)			
Diabetes mellitus	32 (21.3)	15 (18.8)	0.643
Coronary heart disease	28 (18.7)	14 (17.5)	0.818

EG, endoscopic group; CG, craniotomy group; SBP, systolic blood pressure; DBP, diastolic blood pressure; GCS, glasgow coma scale; TC, total cholesterol; TG, triglyceride; LDL-C, low-density lipoprotein cholesterol; HDL-C, high-density lipoprotein cholesterol; BMI, body mass index. Measurement data are expressed as mean ± standard deviation, and were compared using independent sample t-test; count data are expressed as *n* (%), and were compared using *χ*^2^ test.

### 3.2 Comparison of surgical efficacy and postoperative outcomes between the two groups

Comparative analysis of surgical efficacy and postoperative outcomes indicated that the EG exhibited favorable minimally invasive features, while core hematoma evacuation and prognostic outcomes were comparable between groups.

For intraoperative indicators, the average operation time of the EG was (95.3 ± 18.6) min, which was significantly shorter than (192.5 ± 25.4) min of the CG (*P* < 0.001); the average intraoperative blood loss of the EG was (85.6 ± 22.3) mL, which was considerably less than (306.8 ± 35.7) mL of the CG (*P* < 0.001), reflecting the obvious minimally invasive advantage of the endoscopic approach. Regarding hematoma evacuation effect, the average hematoma clearance rate of the EG was (89.2 ± 7.5)%, and that of the CG was (87.6 ± 8.2)%, with no statistically significant difference between the two groups (*P* = 0.148), suggesting that both surgical methods could achieve effective hematoma evacuation.

The postoperative rebleeding rate within 7 days was 4.0% (6/150) in the EG and 5.0% (4/80) in the CG, and there was no significant difference between the two groups (*P* = 0.732). Although the endoscopic approach carries inherent hemostatic challenges related to the narrow working channel, these risks were well managed by experienced operators, and no excess rebleeding was observed. For overall postoperative complications, the total rate was significantly lower in the EG (10.0%, 15/150) than in the CG (28.8%, 23/80) (*P* < 0.001). Specifically, the CG had a higher incidence of postoperative cerebral infarction (3.8% vs. 0.0%, *P* = 0.031), intracranial infection (5.0% vs. 1.3%, *P* = 0.048), pulmonary infection (10.0% vs. 3.3%, *P* = 0.022) and lower extremity deep venous thrombosis (5.0% vs. 1.3%, *P* = 0.048) compared with the EG, which might be related to the larger surgical trauma and longer operation time of the craniotomy approach.

For long-term prognosis, the 3-month favorable prognosis rate was 62.0% (93/150) in the EG and 58.8% (47/80) in the CG, and the unfavorable prognosis rate was 38.0% (57/150) and 41.2% (33/80) respectively, with no statistically significant difference between the two groups (*P* = 0.628). The detailed data of surgical efficacy and postoperative outcomes are summarized in Table 2.

**Table 2:** Comparison of surgical efficacy and postoperative outcomes between the Two groups.

Indicators	EG (*n* = 150)	CG (*n* = 80)	*P*
Operation time (min)	95.3 ± 18.6	192.5 ± 25.4	<0.001
Intraoperative blood loss (mL)	85.6 ± 22.3	306.8 ± 35.7	<0.001
Hematoma clearance rate (%)	89.2 ± 7.5	87.6 ± 8.2	0.148
Postoperative rebleeding (*n*, %)	6 (4.0)	4 (5.0)	0.732
Postoperative cerebral infarction (*n*, %)	0 (0.0)	3 (3.8)	0.031
Intracranial infection (*n*, %)	2 (1.3)	4 (5.0)	0.048
Pulmonary infection (*n*, %)	5 (3.3)	8 (10.0)	0.022
Lower extremity DVT (*n*, %)	2 (1.3)	4 (5.0)	0.048
Total complications (*n*, %)	15 (10.0)	23 (28.8)	<0.001
3-month favorable prognosis (mRS 0–2, *n*, %)	93 (62.0)	47 (58.8)	0.628
3-month unfavorable prognosis (mRS 3–6, *n*, %)	57 (38.0)	33 (41.2)	0.628

EG, endoscopic group; CG, craniotomy group; DVT, deep venous thrombosis; mRS, modified rankin scale. Measurement data are expressed as mean ± standard deviation, and were compared using independent sample t-test; count data are expressed as *n* (%), and were compared using *χ*^2^ test.

### 3.3 Correlation analysis between 3-month favorable prognosis and baseline clinical data

To explore the correlation between 3-month favorable prognosis and preoperative baseline clinical data in patients with basal ganglia HICH, univariate analysis was performed on all baseline parameters of the 230 included patients (favorable prognosis group, *n* = 140; unfavorable prognosis group, *n* = 90). The analysis included demographic data, preoperative neurological function indicators, hematoma volume, hemodynamic indicators, blood lipid profiles and comorbidities, and the statistical methods were selected according to the type of variables (independent sample t-test for measurement data, *χ*^2^ test for count data, and *χ*^2^ trend test for ordered categorical variables with gradient characteristics). On this basis, multivariate Logistic regression analysis was further conducted to screen the independent baseline factors affecting the 3-month favorable prognosis, so as to identify the key preoperative influencing factors for the clinical outcome of patients.

#### 3.3.1 Univariate analysis of correlation between baseline data and 3-month favorable prognosis

The results of univariate analysis indicated that admission GCS score and preoperative hematoma volume were significantly correlated with the 3-month favorable prognosis of patients (all *P* < 0.001), and the ordered categorical *χ*^2^ trend test confirmed that there was a significant linear gradient correlation between the two indicators and prognosis; other baseline parameters including demographic data (age, gender, BMI), preoperative hemodynamic indicators (SBP, DBP), baseline blood lipid profiles (TC, TG, LDL-C, HDL-C) and comorbidities (diabetes mellitus, coronary heart disease) had no significant correlation with the 3-month favorable prognosis (all *P* > 0.05).

For admission GCS score, the favorable prognosis rate showed a significant upward trend with the increase of GCS score: the favorable prognosis rate of the 3∼8 points subgroup was only 23.08% (12/52), that of the 9∼12 points subgroup was 51.09% (47/92), and that of the 13∼15 points subgroup reached 94.19% (81/86), with a statistically significant trend (*P* < 0.001).

For preoperative hematoma volume, the favorable prognosis rate presented a significant downward trend with the increase of hematoma volume: the favorable prognosis rate of the 20∼40 mL subgroup was 82.11% (78/95), that of the 41∼60 mL subgroup was 51.14% (45/88), and that of the 61∼80 mL subgroup was only 36.17% (17/47), with a statistically significant trend (*P* < 0.001).

For other baseline parameters, there were no statistically significant differences in the distribution of gender, age, BMI, preoperative SBP/DBP, blood lipid levels and the incidence of diabetes/coronary heart disease between the favorable prognosis group and the unfavorable prognosis group (all *P* > 0.05), indicating that the above indicators had no obvious correlation with the short-term prognosis of patients in this study population. The detailed results of univariate analysis are shown in Table 3.

**Table 3:** Univariate analysis of correlation between baseline clinical data and 3-month favorable prognosis in basal ganglia HICH patients.

Baseline Parameters	Favorable prognosis group (*n* = 140)	Unfavorable prognosis group (*n* = 90)	*P*
Demographic data			
Gender (male/female, *n*)	85/55	54/36	0.857
Age (years)	61.5 ± 8.6	63.7 ± 8.8	0.086
BMI (kg/m^2^)	24.1 ± 3.0	24.0 ± 2.9	0.831
Preoperative neurological function & disease severity indicators			
Admission GCS score (*n*, %)			<0.001
3∼8 points	12 (8.57)	40 (44.44)	
9∼12 points	47 (33.57)	45 (50.00)	
13∼15 points	81 (57.86)	5 (5.56)	
Preoperative hematoma volume (mL, *n*, %)			<0.001
20∼40 mL	78 (55.71)	17 (18.89)	
41∼60 mL	45 (32.14)	43 (47.78)	
61∼80 mL	17 (12.14)	30 (33.33)	
Preoperative hemodynamic indicators (x ± s)			
SBP (mmHg)	178.2 ± 21.9	181.3 ± 23.7	0.311
DBP (mmHg)	102.3 ± 15.1	104.5 ± 15.6	0.291
Baseline blood lipid indicators (mmol/L, x ± s)			
TC	5.25 ± 1.08	5.28 ± 1.09	0.843
TG	1.88 ± 0.60	1.90 ± 0.63	0.807
LDL-C	3.36 ± 0.88	3.44 ± 0.96	0.535
HDL-C	1.11 ± 0.26	1.09 ± 0.27	0.570
Comorbidities (*n*, %)			
Diabetes mellitus	30 (21.43)	17 (18.89)	0.684
Coronary heart disease	26 (18.57)	16 (17.78)	0.882

HICH, hypertensive intracerebral hemorrhage; GCS, glasgow coma scale; SBP, systolic blood pressure; DBP, diastolic blood pressure; TC, total cholesterol; TG, triglyceride; LDL-C, low-density lipoprotein cholesterol; HDL-C, high-density lipoprotein cholesterol; BMI, body mass index; Measurement data are expressed as mean ± standard deviation, and independent sample t-test is used for comparison; Count data are expressed as *n* (%), and *χ*^2^ test is used for comparison, *χ*^2^ trend test is used for ordered categorical variables.

#### 3.3.2 Multivariate logistic regression analysis of independent baseline factors affecting 3-month favorable prognosis

On the basis of univariate analysis, multivariate Logistic regression analysis was performed with 3-month prognosis (favorable=1, unfavorable=0) as the dependent variable, and all baseline parameters with *P* < 0.1 in univariate analysis (age, admission GCS score, preoperative hematoma volume, preoperative SBP/DBP) and clinically important indicators (gender, BMI, comorbidities) as independent variables. For ordered categorical variables (admission GCS score, preoperative hematoma volume), dummy variables were set with the optimal clinical subgroup as the reference (GCS 13∼15 points as the reference for GCS score, 20∼40 mL as the reference for hematoma volume) to further screen the independent baseline factors affecting the 3-month favorable prognosis of patients.

The results of multivariate Logistic regression analysis showed that admission GCS score and preoperative hematoma volume were the only independent baseline risk factors affecting the 3-month favorable prognosis of patients with basal ganglia HICH (all *P* < 0.05), and other baseline parameters had no significant independent predictive effect on the short-term prognosis of patients (all *P* > 0.05). The detailed regression results are shown in Table 4.

**Table 4:** Multivariate logistic regression analysis of independent baseline factors affecting 3-month favorable prognosis in basal ganglia HICH patients.

Independent Variables	Subgroup/Assignment	OR (95%CI)	*P*
Gender (male=1, female=0)	-	1.085 (0.590∼1.996)	0.784
Age (years, continuous)	-	1.009 (0.986∼1.033)	0.453
BMI (kg/m^2^, continuous)	-	1.017 (0.954∼1.084)	0.597
Admission GCS score (points)	13∼15 points (Ref)	1.000 (Reference)	1.000
	9∼12 points	0.218 (0.096∼0.495)	<0.001
	3∼8 points	0.052 (0.015∼0.182)	<0.001
Preoperative hematoma volume (mL)	20∼40 mL (Ref)	1.000 (Reference)	1.000
	41∼60 mL	0.326 (0.145∼0.732)	0.007
	61∼80 mL	0.105 (0.032∼0.348)	<0.001
Preoperative SBP (mmHg, continuous)	-	1.001 (0.995∼1.007)	0.749
Preoperative DBP (mmHg, continuous)	-	0.997 (0.987∼1.007)	0.548
Diabetes mellitus (yes=1, no = 0)	-	1.277 (0.680∼2.399)	0.438
Coronary heart disease (yes=1, no = 0)	-	1.114 (0.566∼2.195)	0.745
Surgical method (craniotomy=1, endoscopic=0)	-	0.935 (0.521∼1.682)	0.827
Constant	-	67.923 (2.156∼2146.581)	0.009

HICH, hypertensive intracerebral hemorrhage; GCS, glasgow coma scale; SBP, systolic blood pressure; DBP, diastolic blood pressure; BMI, body mass index; OR, odds ratio; CI, confidence interval; Ref, reference group; Dependent variable: 3-month prognosis (favorable=1, unfavorable=0); Total sample size *n* = 230.

##### 3.3.2.1 Admission GCS score

Compared with the reference group (13∼15 points), the risk of unfavorable prognosis in the 9∼12 points subgroup was significantly increased (OR = 0.218, 95%CI: 0.096∼0.495, *P* < 0.001), and the risk of unfavorable prognosis in the 3∼8 points subgroup was further increased (OR = 0.052, 95%CI: 0.015∼0.182, *P* < 0.001), indicating that the decrease of GCS score was an independent risk factor for poor long-term prognosis, and the lower the score, the worse the prognosis.

##### 3.3.2.2 Preoperative hematoma volume

Compared with the reference group (20∼40 mL), the risk of unfavorable prognosis in the 41∼60 mL subgroup was significantly increased (OR = 0.326, 95%CI: 0.145∼0.732, *P* = 0.007), and the risk of unfavorable prognosis in the 61∼80 mL subgroup was significantly higher (OR = 0.105, 95%CI: 0.032∼0.348, *P* < 0.001), suggesting that the increase of hematoma volume was an independent risk factor for poor long-term prognosis, and the larger the volume, the worse the prognosis.

In addition, the regression results also showed that surgical method (endoscopic transfrontal surgery vs. transSylvian-transinsular craniotomy) was not an independent influencing factor for the 3-month favorable prognosis (OR = 0.935, 95%CI: 0.521∼1.682, *P* = 0.827), which further supported that the two surgical methods had comparable core therapeutic efficacy in the treatment of basal ganglia HICH.

### 3.4 Correlation analysis between 3-month favorable prognosis and surgical related indicators

To explore the impact of surgical related indicators on the short-term prognosis of basal ganglia HICH patients, univariate analysis was conducted on all surgical efficacy and postoperative outcome indicators of 230 enrolled patients (favorable prognosis group, *n* = 140; unfavorable prognosis group, *n* = 90) based on Table 2 data, including intraoperative parameters, hematoma evacuation effect, and postoperative adverse events/complications. Independent sample *t*-test was used for continuous measurement data, and *χ*^2^ test for binary count data. On this basis, multivariate Logistic regression analysis was further performed, incorporating the independent baseline prognostic factors (admission GCS score, preoperative hematoma volume) screened in Section 3.3 to correct baseline confounding effects, so as to identify surgical related indicators with independent predictive value for 3-month favorable prognosis.

#### 3.4.1 Univariate analysis of correlation between surgical related indicators and 3-month favorable prognosis

Univariate analysis results indicated that intraoperative blood loss, postoperative cerebral infarction, intracranial infection, pulmonary infection, lower extremity DVT and total postoperative complications were significantly correlated with 3-month favorable prognosis (all *P* < 0.05), while operation time, hematoma clearance rate and postoperative rebleeding had no significant correlation with long-term prognosis (all *P* > 0.05), with detailed results shown in Table 5. For continuous indicators, the average intraoperative blood loss in the favorable prognosis group [(156.8 ± 112.5) ml] was significantly lower than that in the unfavorable prognosis group [(268.3 ± 135.7) ml] (*P* < 0.001), while there were no significant differences in operation time and hematoma clearance rate between the two groups (*P* > 0.05). For binary indicators, the incidence of postoperative rebleeding was similar between the two groups (4.3% vs. 4.4%, *P* = 0.948), while the incidence of all single postoperative complications and total complication rate in the favorable prognosis group were significantly lower than those in the unfavorable prognosis group (all *P* < 0.05), with the total complication rate being 12.1% and 31.1% respectively (*P* < 0.001).

**Table 5:** Univariate analysis of correlation between surgical related indicators and 3-month favorable prognosis in basal ganglia HICH patients.

Surgical Related Indicators	Favorable prognosis group (*n* = 140)	Unfavorable prognosis group (*n* = 90)	*P*
Intraoperative operation parameters			
Operation time (min)	128.5 ± 45.6	142.3 ± 52.1	0.059
Intraoperative blood loss (mL)	156.8 ± 112.5	268.3 ± 135.7	<0.001
Hematoma evacuation effect			
Hematoma clearance rate (%)	88.9 ± 7.8	87.5 ± 8.1	0.210
Postoperative adverse events (*n*, %)			
Postoperative rebreeding	6 (4.3)	4 (4.4)	0.948
Postoperative complications (*n*, %)			
Postoperative cerebral infarction	1 (0.7)	2 (2.2)	0.045
Intracranial infection	2 (1.4)	4 (4.4)	0.039
Pulmonary infection	6 (4.3)	10 (11.1)	0.014
Lower extremity DVT	2 (1.4)	4 (4.4)	0.039
Total complications	17 (12.1)	28 (31.1)	<0.001

HICH, hypertensive intracerebral hemorrhage; DVT, deep venous thrombosis; Measurement data are expressed as mean ± standard deviation and compared by independent sample *t*-test; count data are expressed as *n* (%) and compared by *χ*^2^ test; Total sample size *n* = 230, with 140 cases in the favorable prognosis group (mRS 0–2) and 90 cases in the unfavorable prognosis group (mRS 3–6).

#### 3.4.2 Multivariate logistic regression analysis of independent surgical factors affecting 3-month favorable prognosis

With 3-month prognosis (favorable=1, unfavorable=0) as the dependent variable, multivariate Logistic regression analysis was performed by including surgical related indicators with *P* < 0.1 in univariate analysis and independent baseline prognostic factors (admission GCS score, preoperative hematoma volume, set as dummy variables with optimal subgroups as reference), and the results are shown in Table 6. After adjusting for baseline factors, the results demonstrated that intraoperative blood loss and postoperative pulmonary infection were independent surgical risk factors for unfavorable 3-month prognosis (both *P* < 0.05), while other surgical indicators such as operation time, postoperative cerebral infarction, intracranial infection, lower extremity DVT and total complications had no significant independent predictive effect (all *P* > 0.05). Specifically, intraoperative blood loss as a continuous variable was an independent risk factor (OR = 0.997, 95%CI: 0.995∼0.999, *P* = 0.008), with each 1 mL increase in blood loss associated with a 0.3% increase in the risk of unfavorable prognosis; postoperative pulmonary infection significantly increased the risk of poor prognosis (OR = 3.258, 95%CI: 1.186∼8.965, *P* = 0.022), with patients with pulmonary infection having a 3.258-fold higher risk of short-term unfavorable prognosis than those without. In addition, the regression results confirmed that admission GCS score and preoperative hematoma volume still had significant independent predictive effects on prognosis after incorporating surgical related indicators (all *P* < 0.001), supporting the core role of baseline indicators in prognostic prediction.

**Table 6:** Multivariate logistic regression analysis of independent surgical factors affecting 3-month favorable prognosis in basal ganglia HICH patients.

Independent Variables	Subgroup/Assignment	OR (95%CI)	***P***
Baseline independent factors (for correction)			
Admission GCS score (points)	13∼15 points (Ref)	1.000 (Reference)	1.000
	9∼12 points	0.226 (0.098∼0.523)	<0.001
	3∼8 points	0.055 (0.016∼0.189)	<0.001
Preoperative hematoma volume (mL)	20∼40 mL (Ref)	1.000 (Reference)	1.000
	41∼60 mL	0.333 (0.146∼0.759)	0.008
	61∼80 mL	0.113 (0.033∼0.385)	<0.001
Surgical related indicators			
Operation time (min, continuous)	-	1.001 (0.997∼1.005)	0.617
Intraoperative blood loss (mL, continuous)	-	0.997 (0.995∼0.999)	0.008
Postoperative rebleeding (yes=1, no = 0)	-	1.025 (0.301∼3.498)	0.980
Postoperative cerebral infarction (yes=1, no = 0)	-	2.448 (0.298∼20.125)	0.348
Intracranial infection (yes=1, no = 0)	-	2.193 (0.605∼7.942)	0.231
Pulmonary infection (yes=1, no = 0)	-	3.258 (1.186∼8.965)	0.022
Lower extremity DVT (yes=1, no = 0)	-	2.193 (0.605∼7.942)	0.231
Total complications (yes=1, no = 0)	-	1.764 (0.825∼3.772)	0.138
Constant	—	53.782 (1.896∼1526.358)	0.016

HICH, hypertensive intracerebral hemorrhage; GCS, glasgow coma scale; DVT, deep venous thrombosis; OR, odds ratio; CI, confidence interval; Ref, reference group; The dependent variable is 3-month prognosis (favorable=1, unfavorable=0) with total sample size *n* = 230; The model is adjusted for admission GCS score and preoperative hematoma volume; Constant is the regression model intercept, a necessary mathematical parameter for calibration with no direct clinical interpretability.

### 3.5 Assessment results of sylvian fissure anatomical cognition

#### 3.5.1 Improvement of sylvian fissure anatomical theory score

Before clinical practice, the average score of Sylvian fissure anatomical theory assessment of the 8 young doctors was (65.3 ± 5.9) points; at 6 months after practice, the average theory score was significantly improved to (89.4 ± 4.1) points, with a statistically significant difference in the comparison before and after practice (*P* < 0.001) (Table 7). The assessment paper was detailed in Supplementary Material 1. This exploratory observational analysis indicated that participation in transSylvian-transinsular craniotomy practice may be associated with improved mastery of Sylvian fissure anatomical theoretical knowledge and operative skills, especially the application ability of theoretical knowledge in clinical operative scenarios. The detailed individual preoperative and postoperative scores of each young doctor are presented in Supplementary Table S1.

**Table 7:** Comparison of preoperative and postoperative anatomical cognition assessment scores of young doctors in the craniotomy group.

Assessment type	Preoperative score	Postoperative score	*P*
Anatomical theory assessment (full score 100)	65.3 ± 5.9	89.4 ± 4.1	<0.001
Operative performance assessment (full score 100)	62.4 ± 7.0	86.8 ± 4.7	<0.001

The anatomical theory assessment was conducted with a closed-book written test, and the detailed assessment paper was in Supplementary Material 1; the operative performance assessment was completed by on-site surgical observation and intraoperative operation video review, and the detailed assessment method was in Supplementary Material 2. The assessment was carried out by 3 senior neurosurgeons (≥10 years of clinical experience), and the final score was the average of the three examiners’ scores.

#### 3.5.2 Improvement of sylvian fissure operative practice skill score

The average score of Sylvian fissure operative practice skill assessment of young doctors before practice was (62.4 ± 7.0) points, which was at a medium level, and the main deficiencies were reflected in the fine separation of Sylvian fissure and the protection of surrounding important structures; after 6 months of clinical practice, the average skill score was increased to (86.8 ± 4.7) points, and the comparison before and after practice was statistically significant (*P* < 0.001) (Table 7). The assessment method was detailed in Supplementary Material 2. All young doctors showed obvious progress in the key operative steps related to Sylvian fissure, and the operational standardization and proficiency were significantly improved. The detailed individual preoperative and postoperative scores of each young doctor are presented in Supplementary Table S1.

### 3.6 Illustrative cases

#### 3.6.1 Case 1: transSylvian-transinsular hematoma evacuation

A 48-year-old male presented to the emergency department with a 2-hour history of acute-onset headache followed by impaired consciousness, with no prior traumatic history. On admission, the patient was in a coma with a GCS score of 7 (E1V2M4). Neurological examination revealed a motor strength of Grade 0/5 in the left extremity. Non-contrast cranial CT demonstrated an intracerebral hemorrhage in the right basal ganglia with rupture into the lateral ventricle; CTA of the cerebral vasculature excluded underlying vascular malformations and intracranial aneurysms. The hematoma volume was quantified as 62 mL via 3D Slicer volumetric reconstruction, presenting as extensive and irregular with a 10 mm midline shift. In view of the above findings, emergency hematoma evacuation was performed via a transSylvian-transinsular craniotomy. Postoperative CT confirmed a 96% hematoma evacuation rate, with no new-onset neurological deficits identified. The patient was discharged on postoperative day 25 for outpatient rehabilitation, with a GCS score of 11 (E3V3M5) at the time of discharge (Figure 4).

#### 3.6.2 Case 2: endoscopic transfrontal hematoma evacuation

A 56-year-old female was admitted to the emergency department with a 1-hour history of sudden-onset right hemiparesis and projectile vomiting, with no antecedent head trauma. On admission, she was somnolent with a GCS score of 12 (E3V4M5). Neurological examination revealed a motor strength of Grade 1/5 in the right upper extremity and Grade of 2/5 in the right lower extremity. Non-contrast cranial CT showed intracerebral hemorrhage in the left basal ganglia with rupture into the left lateral ventricle; CTA excluded underlying vascular malformations and aneurysms. The hematoma volume was measured as 35 mL using 3D Slicer reconstruction. Given the large, regular ovoid hematoma associated with a 6 mm midline shift, emergency endoscopic transfrontal hematoma evacuation was performed. Postoperative CT confirmed an 83% hematoma clearance rate with no new neurological deficits. The patient was discharged on postoperative day 8 for further rehabilitation, with a fully alert mental status, right upper extremity motor Grade of 3/5 and right lower extremity a motor strength Grade of 4/5 at discharge (Figure 5).

## 4 Discussion

### 4.1 Comparative efficacy of the two surgical methods for basal ganglia HICH

Basal ganglia HICH is a major global neurological burden, with high disability and mortality rates that impose substantial healthcare pressure worldwide (1, 5). Timely and effective hematoma evacuation is the core measure to improve prognosis, as emphasized in both American and European clinical guidelines (6, 19). This study indicated that endoscopic transfrontal surgery and transSylvian-transinsular craniotomy had comparable core therapeutic efficacy for basal ganglia HICH, with no significant differences in hematoma clearance rate, postoperative rebleeding rate, and 3-month favorable prognosis rate between the two groups—findings consistent with the conclusions of multiple recent studies (8, 16, 20).

Wang et al. compared endoscopic surgery and craniotomy for hypertensive putamen hemorrhage and suggested that both approaches achieved similar clinical efficacy in terms of functional outcomes, which aligns with our findings (20). Potts et al. also reported that transSylvian-transinsular approaches achieved complete resection in 87.5% of arteriovenous malformations and 95% of cavernous malformations, with low permanent neurological morbidity, supporting the safety and effectiveness of this approach for deep-seated lesions involving the insula and basal ganglia (11). Additionally, Jianwei et al. demonstrated that the transSylvian-transinsular microsurgical approach for hypertensive putaminal hematomas could obtain satisfactory hematoma evacuation effects, further supporting the clinical value of this craniotomy method (10).

Endoscopic surgery showed prominent minimally invasive advantages, including significantly shorter operation time, less intraoperative blood loss, and lower total postoperative complication rate—attributed to small craniotomy, simplified surgical steps, and reduced tissue damage. This is consistent with the previous studies, which indicated that endoscopic surgery for supratentorial hypertensive intracerebral hemorrhage had obvious advantages in reducing surgical trauma and postoperative complications compared with craniotomy (7). A 2025 meta-analysis further suggested that neuroendoscopic surgery for HICH is associated with fewer postoperative complications and shorter hospital stays than conventional craniotomy (8). In contrast, craniotomy involves larger surgical trauma and longer operation time due to Sylvian fissure separation, leading to a higher incidence of postoperative complications such as cerebral infarction, pulmonary infection, and intracranial infection. Tanriover et al. conducted a detailed microsurgical anatomical study on the insula and Sylvian fissure, pointing out that the transSylvian approach involves complex vascular and anatomical structures (e.g., middle cerebral artery branches and deep venous system), and the long operation time and extensive tissue dissection may increase the risk of complications—consistent with the results of this study (12).

### 4.2 Independent prognostic factors for basal ganglia HICH

Univariate and multivariate analyses indicated that admission GCS score and preoperative hematoma volume were the only independent baseline risk factors for 3-month unfavorable prognosis of basal ganglia HICH patients, which is in line with domestic and foreign clinical consensus and guideline recommendations (6, 19). Tahara et al. analyzed a Japanese nationwide database and found that preoperative GCS score and hematoma volume were important predictors of prognosis in patients with spontaneous intracerebral hemorrhage, which is consistent with our results (14). Van et al. also reported in a systematic review and meta-analysis that hematoma volume and initial neurological function are key determinants of functional outcome in HICH patients (21).

GCS score directly reflects the degree of preoperative neurological injury and consciousness state, and lower scores indicate poorer brainstem function and weaker recovery potential. Larger hematoma volume causes more severe compression of surrounding brain tissue and internal capsule, easily leading to severe cerebral edema, increased intracranial pressure, and secondary brain injury—resulting in difficult recovery of neurological function (22). The two indicators have obvious synergistic effects: patients with small hematoma volume combined with high GCS score have the best prognosis, suggesting that clinical doctors should conduct comprehensive evaluation and formulate individualized treatment plans based on these two core indicators, as recommended in the AHA/ASA guidelines (23, 24).

Surgical related indicators also affect short-term prognosis: intraoperative blood loss and postoperative pulmonary infection were the core independent surgical risk factors after correcting baseline confounding factors. Every 1 mL increase in intraoperative blood loss was associated with an increased risk of unfavorable prognosis, indicating that minimizing blood loss through standardized surgery is an important measure to improve prognosis. Xu et al. reported similar findings in a multicenter randomized controlled trial (MISICH), which found that intraoperative blood loss is an independent risk factor for poor prognosis in HICH patients treated with minimally invasive surgery (25). Postoperative pulmonary infection increased the risk of poor short-term prognosis by more than 3 times, which is an independent adverse event beyond baseline conditions—possibly related to postoperative bed rest, inadequate respiratory tract management, and reduced respiratory function in patients. Yu et al. also pointed out that postoperative infections (including pulmonary infection) are important preventable factors affecting the prognosis of HICH patients, and active preventive strategies can improve clinical outcomes (26).

Univariate analysis found that cerebral infarction, intracranial infection, and other complications were correlated with prognosis, but they lost independence after baseline adjustment—mostly related to poor preoperative baseline conditions (e.g., low GCS score and large hematoma volume). Operation time, hematoma clearance rate, and postoperative rebleeding had no significant correlation with short-term prognosis, because both surgical methods achieved effective hematoma evacuation (consistent with the high clearance rate reported by Li et al., in a comparison of three surgical methods) and surgeons’ rich experience ensured effective hemostasis and reasonable operation time control (27).

### 4.3 Clinical teaching value of transSylvian-transinsular craniotomy

The primary innovation of this study lies in that it is the first attempt to conduct an exploratory observational analysis to investigate and quantitatively assess the potential educational value of transSylvian-transinsular craniotomy for improving young doctors’ anatomical cognition of the Sylvian fissure. It should be noted that this part is not a rigorously designed controlled trial, and relevant findings need to be interpreted with caution. The Sylvian fissure has a complex anatomical structure with abundant blood vessels and membranous tissues, which is a key and difficult point for young neurosurgeons’ learning (12, 13, 28). Tayebi et al. emphasized that Sylvian fissure splitting is a core microsurgical skill that requires a solid understanding of arachnoidal anatomy and practical experience, which is often lacking in traditional anatomical teaching (29).

Traditional teaching is mainly based on theoretical learning and specimen anatomy, lacking intuitive clinical practice cognition, leading to difficulties in accurate identification and separation of the Sylvian fissure in clinical work. This study exploratively observed that participation in transSylvian-transinsular craniotomy was associated with significant improvement in both theoretical and practical scores related to Sylvian fissure anatomy. It should be noted that this improvement may also be related to the natural learning curve, continuous training, and clinical experience accumulation. Nevertheless, this approach may provide a valuable practical opportunity for young neurosurgeons to understand Sylvian fissure anatomy. Given the lack of a control group and validated scales, this part should be considered as an exploratory teaching observation rather than a conclusive evidence. This is consistent with the educational value of anatomical practice emphasized in microsurgical anatomy studies and the role of simulation and practical training in neurosurgical education (12, 30, 31). Muhammad et al. also reported that hands-on microsurgical dissection of the Sylvian fissure significantly improved young neurosurgeons’ technical proficiency, which supports the teaching value of the transSylvian-transinsular approach demonstrated in our study (13).

### 4.4 Clinical implications and surgical selection strategy

Based on the study results and combined with existing guidelines and evidence (9, 16, 24), a personalized surgical selection strategy for basal ganglia HICH is proposed: endoscopic transfrontal surgery is the first choice for most patients, especially for the elderly with poor general condition, high surgical risk, and hematoma limited to the basal ganglia without extending to the insula/Sylvian fissure. This approach can reduce trauma and improve surgical safety by virtue of its minimally invasive advantages, which is supported by the study showing that minimally invasive endoscopic surgery for acute subdural hematoma had better safety than craniotomy (32), and a recent study suggesting that neuroendoscopy is superior to conventional surgery in the treatment of HICH in terms of safety and efficacy (9).

TransSylvian-transinsular craniotomy is suitable for specific patients with hematoma extending to the insula/Sylvian fissure or combined with arachnoid adhesion—this approach can achieve thorough hematoma evacuation through the natural anatomical space and avoid secondary brain tissue damage caused by blind aspiration of the endoscopic approach. Potts et al. also pointed out that transSylvian-transinsular approaches are more suitable for lesions involving the insula and deep-seated structures, as they can better preserve the overlying eloquent cortex (11). Alberio et al. reported similar findings in a study of minimally invasive management of supratentorial lobar hemorrhages, emphasizing that anatomical approach selection based on hematoma location is crucial for improving outcomes (15).

In clinical treatment, it is necessary to take preoperative GCS score and hematoma volume as key evaluation indicators (22), reduce intraoperative blood loss through standardized surgical operation (25), and strengthen postoperative respiratory tract management and pulmonary infection prevention and control—these are the key links to improve short-term prognosis. In addition, this craniotomy can be used as a key practical teaching project to cultivate young doctors’ Sylvian fissure anatomical cognition, which is in line with the trend of emphasizing practical skills training in neurosurgical education (13, 31).

### 4.5 Study limitations and future research directions

This study has certain limitations: first, this was a retrospective observational study without randomization, so selection bias may exist despite matching and balanced baseline characteristics—most of the existing high-quality studies on HICH surgical treatment adopt multi-center designs to improve the reliability and generalizability of results. Second, the evaluation of Sylvian fissure anatomical cognition was exploratory, with a small sample of young doctors, no control group, no randomization or blinding, and unvalidated assessment scales. As such, the relevant conclusions about the surgical approach's teaching value have limited evidence strength and cannot be widely generalized. Further rigorous controlled trials are required to verify these findings in the future. The observed score improvement may also be affected by the natural learning curve, repeated training, or enhanced supervision rather than the specific effect of the surgical approach itself. Third, the long-term impact of improved anatomical cognition on their surgical skills and clinical work needs further observation. Future studies will collaborate with multiple centers to expand the sample size of both HICH patients and young participating doctors, reducing selection bias through prospective design and standardized data collection. We also plan to conduct long-term follow-up of young doctors, tracking changes in their surgical complication rates, operation time, and patient prognostic outcomes to quantitatively verify the sustained educational value of the transSylvian-transinsular approach.

## 5 Conclusion

Endoscopic transfrontal surgery and transSylvian-transinsular craniotomy have comparable core efficacy for basal ganglia HICH. Endoscopic surgery has distinct minimally invasive advantages, with reduced operation time, less intraoperative blood loss and fewer postoperative complications. As an exploratory observational analysis, this work suggests that transSylvian-transinsular craniotomy may offer potential clinical teaching value for improving young doctors’ anatomical cognition of the Sylvian fissure. Preoperative GCS score and hematoma volume appear to be independent baseline prognostic factors for short-term outcomes, while intraoperative blood loss and postoperative pulmonary infection are core independent surgical risk factors. Clinically, the surgical approach should be selected based on the patient's specific condition, operator's experience and hospital equipment.

## Data Availability

The original contributions presented in the study are included in the article/Supplementary Material, further inquiries can be directed to the corresponding author.
