# Supplementary material for: Retrospective clinical study of endoscopic transfrontal approach vs. transSylvian-transinsular craniotomy for hypertensive intracerebral hemorrhage in basal ganglia: efficacy comparison and value of anatomical cognition of Sylvian fissure
Source: Front Surg. 2026 Jun 18;13:1860820. doi: 10.3389/fsurg.2026.1860820 (PMC13325627; doi:10.3389/fsurg.2026.1860820)
Supplement: Supplementary file 1 [file Supplementaryfile1.docx]

**Supplementary Material 1:**

**Sylvian Fissure Anatomical Theoretical Assessment Paper**

**Part 1: Single-Choice Questions (10 Questions, 5 Points Each, Total 50 Points)**

1. Which of the following is the correct composition of the superficial part of the Sylvian fissure? ( )
2. Stem + anterior horizontal ramus + anterior ascending ramus + posterior ramus
3. Stem + frontoparietal ramus + temporal ramus + occipital ramus
4. Stem + superficial ramus + deep ramus + opercular ramus
5. Stem + cisternal ramus + opercular ramus + insular ramus
6. The mean distance from the origin of the most lateral lenticulostriate artery (LSA) to the insular apex is approximately ( )

A. 8.6 mmB. 14.6 mmC. 20.6 mmD. 26.6 mm

1. Which segment of the middle cerebral artery (MCA) is mainly responsible for supplying the anterior, middle, and posterior short gyri of the insula? ( )
2. M1 segment (sphenoidal segment)
3. M2 segment (insular segment) superior trunk
4. M2 segment (insular segment) inferior trunk

D. M3 segment (opercular segment)

1. The "limen recess" between the medial border of the limen insulae and the lateral limit of the anterior perforated substance is characterized by ( )
2. Rich in perforating arteries
3. Devoid of perforating arteries
4. Directly connected to the deep middle cerebral vein (dMCV)

D. The origin of the superficial Sylvian vein (SSV)

1. Which vein is most commonly connected to the superficial Sylvian vein (SSV) among the insular veins? ( )
2. Anterior insular vein
3. Precentral insular vein
4. Central insular vein

D. Posterior insular vein

1. When splitting the Sylvian fissure, the "paperknife technique" refers to dissection in which direction? ( )
2. Superficial-to-deep + anterior-to-posterior
3. Deep-to-superficial + posterior-to-anterior
4. Medial-to-lateral + superior-to-inferior

D. Lateral-to-medial + inferior-to-superior

7. The deep portion of the Sylvian fissure is divided into two compartments, which are ( )

A. Superficial opercular compartment + deep opercular compartment

B. Sphenoidal compartment + operculoinsular compartment

C. Cisternal compartment + insular compartment

D. Frontal compartment + temporal compartment

8. Which cortical artery most commonly supplies the middle short gyrus of the insula? ( )

A. Orbitofrontal artery B. Precentral artery C. Central artery D. Angular artery

1. The mean width of the limen insulae is approximately ( )

A. 15.3 mm B. 21.36 mm C. 25.0 mm D. 34.0 mm

1. During the transsylvian-transinsular approach, the anterior approach mainly exposes which structures of the insula? ( )
2. Limen insulae + short gyri
3. Circular sulcus + long gyri
4. Central insular sulcus + anterior long gyrus

D. Inferior limiting sulcus + posterior long gyrus

**Part 2: Multiple-Choice Questions (4 Questions, 5 Points Each, Total 20 Points; *Multiple Selections Allowed, Only Full Correct Answers Score, No Partial Points*)**

1. Which of the following belong to the membranous structures related to the Sylvian fissure? ( )
2. Outer arachnoid membrane (OAM)
3. Lateral Sylvian membrane (LSM)
4. Intermediate Sylvian membrane (ISM)

D. Proximal Sylvian membrane (PSM)

2. The arterial supply of the insula involves which segments or branches of the MCA? ( )

A. M1 segment (sphenoidal segment)

B. M2 segment (insular segment) superior trunk

C. M2 segment (insular segment) inferior trunk

D. Early branches of the MCA

3. Which complications may be caused by improper sacrifice of the superficial Sylvian vein (SSV) during Sylvian fissure dissection? ( )

A. Venous infarction B. Cerebral edema C. Facial palsy D. Seizures

4. The key points of venous-preserving Sylvian fissure dissection include ( )

A. Identifying the "microvascular Sylvian fissure" as the dissection plane

B. Using the "denude technique" to peel off the arachnoid around the vein

C. Sacrificing small tributaries of the SSV to expand the surgical corridor

D. Minimizing brain retraction to avoid venous compression

**Part 3: Essay Questions (3 Questions, 10 Points Each, Total 30 Points)**

1. Describe the topographic anatomy of the Sylvian fissure, including its superficial rami, deep compartments, and key anatomical landmarks.
2. Explain the arterial supply pattern of the insula, focusing on the distribution of the superior and inferior trunks of the MCA M2 segment and the common cortical artery branches involved.
3. Summarize the surgical precautions for Sylvian fissure splitting to prevent complications, combining venous preservation techniques and anatomical characteristics.

**Answer Key and Scoring Standards**

**Part 1: Single-Choice Questions (5 Points Each)**

1. A (Ref 15 in the manuscript: The superficial part of the Sylvian fissure is composed of a stem and several rami, including anterior horizontal, anterior ascending, and posterior rami)
2. B (Ref 15 in the manuscript: The distance between the origin of the most lateral LSA and the insular apex was 14.6 mm)
3. B (Ref 15 in the manuscript: The superior trunk of the M2 segment supplies the anterior, middle, and posterior short gyri of the insula)
4. B (Ref 15 in the manuscript：The limen recess is devoid of perforating arteries)
5. B (Ref 15 in the manuscript: The precentral insular vein is most commonly connected to the SSV)
6. B (Ref 26 in the manuscript: The "paperknife technique" is dissection in a deep-to-superficial and posterior-to-anterior fashion)
7. B (Ref 15 in the manuscript: The deep portion of the Sylvian fissure is divided into sphenoidal and operculoinsular compartments)
8. B (Ref 15 in the manuscript: The precentral artery most commonly supplies the middle short gyrus)
9. B (Ref 15 in the manuscript: The mean width of the limen insulae is 21.36 mm)
10. A (Ref 15 in the manuscript: The anterior transsylvian-transinsular approach exposes the limen insulae and short gyri)

**Part 2: Multiple-Choice Questions (5 Points Each)**

1. ABCD (Ref 27 in the manuscript: The Sylvian fissure is related to OAM, LSM, ISM, and PSM)
2. ABCD (Ref 15 in the manuscript: The insular arterial supply involves M1, M2 superior/inferior trunks, and early MCA branches)
3. ABCD (Ref 26 in the manuscript: Sacrificing SSV may cause venous infarction, edema, facial palsy, and seizures)
4. ABD (Ref 26 in the manuscript: Venous-preserving techniques include identifying the correct plane, denude technique, and minimizing retraction; sacrificing tributaries is not recommended)

**Part 3: Essay Questions (10 Points Each)**

1. **Scoring Standards(Ref 15 and 27 in the manuscript)**

1.1 Correct description of superficial rami (stem + anterior horizontal/anterior ascending/posterior rami; mention of frontoorbital ramus in 65% of hemispheres, 3 points);

1.2 Correct division of deep compartments (sphenoidal compartment [proximal to limen area] + operculoinsular compartment [deep to superficial rami, including opercular and insular clefts], 2 points);

1.3 Key landmarks (limen insulae, insular apex [most lateral projecting point] / pole [anteroinferior edge], central insular sulcus [divides insula into anterior short gyri and posterior long gyri], circular limiting sulcus [anterior/superior/inferior parts], 3 points);

1.4 Logical and accurate expression (no anatomical errors, 2 points).

1. **Scoring Standards (Ref 15 in the manuscript)**

2.1 Superior trunk supply area (anterior/middle/posterior short gyri, insular apex, anterior limiting sulcus, short insular sulci; no overlap with inferior trunk supply, 3 points);

2.2 Inferior trunk supply area (posterior long gyrus, inferior limiting sulcus, limen area; 90% supply to inferior limiting sulcus and limen insulae, 3 points);

2.3 Common cortical arteries (prefrontal artery [supplies anterior short gyrus], precentral artery [middle short gyrus], central artery [central insular sulcus, posterior short gyrus], angular artery [posterior long gyrus], 2 points);

2.4 Clear classification and no omissions (mention of "mixed zone" [central insular sulcus/anterior long gyrus] supplied by either trunk, 2 points).

1. **Scoring Standards (Ref 26 and 27 in the manuscript)**

3.1 Venous preservation (identify "microvascular Sylvian fissure" as dissection plane between frontal/temporal vessels; use "denude technique" to peel arachnoid around SSV for stretchability; avoid sacrificing SSV and tributaries to prevent venous infarction/edema, 3 points);

3.2 Vascular protection (preserve MCA M2/M3 segments and en passage arteries; avoid injury to LSA [origin 14.6 mm from insular apex] and large insular perforating arteries [arise from central/angular arteries], 3 points);

3.3 Anatomical guidance (use pars triangularis/rami as landmarks; adopt "paperknife technique" [deep-to-superficial, posterior-to-anterior] for tight opercular adhesion; minimize brain retraction to avoid cortical contusion, 2 points);

3.4 Practical and feasible suggestions (assess SSV course preoperatively; dissect along arachnoid planes to reduce tissue damage, 2 points).
