# Supplementary material for: Retrospective clinical study of endoscopic transfrontal approach vs. transSylvian-transinsular craniotomy for hypertensive intracerebral hemorrhage in basal ganglia: efficacy comparison and value of anatomical cognition of Sylvian fissure
Source: Front Surg. 2026 Jun 18;13:1860820. doi: 10.3389/fsurg.2026.1860820 (PMC13325627; doi:10.3389/fsurg.2026.1860820)
Supplement: Supplementary file 2 [file Supplementaryfile2.docx]

**Supplementary Material 2**.

**Detailed Scoring Standards for Sylvian Fissure Operative Performance Assessment (Full Score: 100 Points)**

| **Evaluation Dimension** | **Full Score** | **Scoring Standards (Ref 14, 15, 26, 27 in the manuscript)** |
| --- | --- | --- |
| Accurate identification of Sylvian fissure anatomical structures | 30 points | **27~30 points:** 1. Accurately and quickly identifies all key superficial structures: Sylvian fissure stem, anterior horizontal/anterior ascending/posterior rami (and frontoorbital ramus if present), superficial Sylvian vein (SSV) and its tributaries (2 points); 2. Precisely locates deep key landmarks: limen insulae, insular apex/pole, central insular sulcus, circular limiting sulcus (anterior/superior/inferior parts) (3 points); 3. Clearly distinguishes MCA segments (M1/M2/M3) and their branches (superior/inferior trunks of M2 segment) (3 points); 4. Identifies lenticulostriate artery (LSA) origin (14.6 mm from insular apex on average) and limen recess (devoid of perforating arteries) (2 points); 5. No misidentification or hesitation during the whole process, and can use anatomical markers to guide surgical path selection (2 points). **18~26 points:** 1. Accurately identifies main superficial structures (rami, SSV) but hesitates slightly in recognizing frontoorbital ramus or small tributaries (2 points); 2. Masters core deep landmarks (limen insulae, central insular sulcus) but has minor errors in describing the location of insular apex/pole (3 points); 3. Distinguishes main MCA segments (M1/M2/M3) but is unclear about the division of M2 superior/inferior trunks (3 points); 4. Identifies LSA but ignores limen recess (2 points); 5. No misidentification affecting surgical progress (2 points). **0~17 points:** 1. Misidentifies key superficial structures (e.g., confuses anterior ascending ramus with anterior horizontal ramus) (2 points); 2. Fails to recognize core deep landmarks (limen insulae or central insular sulcus) (3 points); 3. Cannot distinguish MCA segments or confuses M2/M3 segments (3 points); 4. Fails to identify LSA leading to potential injury risk (2 points); 5. Misidentification causes surgical stagnation or adjustment (2 points). |
| Standardization of Sylvian fissure separation techniques | 30 points | **27~30 points:** 1. Strictly selects dissection plane: identifies "microvascular Sylvian fissure" (between frontal/temporal vessels) as the separation path, no injury to frontal/temporal cortical vessels (4 points); 2. Adopts correct dissection sequence: uses "paperknife technique" (deep-to-superficial + posterior-to-anterior) for tight opercular adhesion, avoids forced separation (3 points); 3. Standard operation movements: uses microscissors and forceps to split arachnoid membrane in small increments, moderate strength, smooth separation process (3 points); 4. Venous preservation technique: uses "denude technique" to peel arachnoid around SSV for stretchability, no compression or traction injury to SSV (3 points); 5. Complete preservation of membranous structures: retains integrity of outer arachnoid membrane and lateral Sylvian membrane, no excessive tearing (2 points). **18~26 points:** 1. Basically selects correct dissection plane but has minor deviation, no vascular injury (4 points); 2. Dissection sequence is basically standard but uses superficial-to-deep separation in partial areas, no adverse effects (3 points); 3. Operation movements are mostly standard but has occasional rough traction (3 points); 4. Preserves SSV but does not use "denude technique", slight adhesion between vein and brain tissue (3 points); 5. Minor damage to membranous structures, no impact on surgical field exposure (2 points). **0~17 points:** 1. Dissection plane is wrong (e.g., crosses SSV directly), leading to venous tributary injury (4 points); 2. Forced separation in superficial-to-deep direction, causing opercular cortical contusion (3 points); 3. Rough operation, excessive traction leading to arachnoid tearing and bleeding (3 points); 4. Sacrifices SSV or causes venous congestion due to improper operation (3 points); 5. Severe damage to membranous structures, affecting exposure of deep structures (2 points). |
| Protection of vital structures | 20 points | **18~20 points:** 1. MCA and branches: avoids compression of M2/M3 segments and en passage arteries, no spasm or injury (3 points); 2. Perforating arteries: strictly protects LSA and large insular perforating arteries (originating from central/angular arteries), no accidental coagulation or cutting (3 points); 3. Insular cortex: minimizes transgression of insular cortex, no excessive resection of short/long gyri (2 points); 4. Venous system: completely preserves SSV and deep middle cerebral vein (dMCV), no thrombosis or outflow obstruction (3 points); 5. No iatrogenic injury to surrounding nerves or brain tissue during the whole process (1 point). **12~17 points:** 1. Basically preserves MCA main trunks but causes slight spasm of small branches (3 points); 2. Protects LSA but ignores small insular perforating arteries (3 points); 3. Minor transgression of insular cortex, no clinical damage (2 points); 4. Preserves SSV main trunk but has minor injury to small tributaries (3 points); 5. No severe vital structure injury (1 point). **0~11 points:** 1. Injury to MCA branches leading to intraoperative bleeding or postoperative infarction (3 points); 2. Accidental injury to LSA causing basal ganglia ischemia (3 points); 3. Excessive resection of insular cortex leading to neurological deficit risk (2 points); 4. Damage to SSV leading to venous infarction (3 points); 5. Iatrogenic injury to surrounding nerves (e.g., optic nerve) or brain tissue (1 point). |
| Surgical operation coordination | 20 points | **18~20 points:** 1. Team coordination: cooperates with assistants and instrument nurses seamlessly, accurately expresses surgical demands (e.g., retractor direction, instrument selection) (3 points); 2. Instrument use: reasonable selection of microscissors, forceps, and suction tube, no frequent instrument replacement (3 points); 3. Step connection: smooth transition between dissection, hemostasis, and exposure steps, no unnecessary pauses (2 points); 4. Adaptability: timely adjusts operation strategy according to anatomical variations (e.g., presence of frontoorbital ramus, SSV course variation) (3 points); 5. No impact on operation progress due to coordination issues (1 point). **12~17 points:** 1. Basic team coordination but has minor delays in expressing demands (3 points); 2. Instrument selection is mostly reasonable but has occasional inappropriate use (3 points); 3. Step connection is basically smooth but has short pauses in partial links (2 points); 4. Can adjust strategy for common anatomical variations but hesitates in rare variations (3 points); 5. Minor impact on operation progress (1 point). **0~11 points:** 1. Poor team coordination, unclear expression leading to wrong instrument handover (3 points); 2. Frequent instrument replacement, improper use of tools leading to tissue damage (3 points); 3. Disrupted step connection, long pauses or repeated operations (2 points); 4. Cannot adapt to anatomical variations, leading to surgical plan adjustment (3 points); 5. Severe delay in operation progress (1 point). |
