# Supplementary material for: Retrospective clinical study of endoscopic transfrontal approach vs. transSylvian-transinsular craniotomy for hypertensive intracerebral hemorrhage in basal ganglia: efficacy comparison and value of anatomical cognition of Sylvian fissure
Source: Front Surg. 2026 Jun 18;13:1860820. doi: 10.3389/fsurg.2026.1860820 (PMC13325627; doi:10.3389/fsurg.2026.1860820)
Supplement: Supplementary file 3 [file Table1.docx]

**Supplementary Table S1** Individual Preoperative and Postoperative Anatomical Cognition Assessment Scores of 8 Young Doctors in the Craniotomy Group

| **Doctor ID** | **Anatomical theory assessment (full score 100)** | | **Operative performance assessment (full score 100)** | |
| --- | --- | --- | --- | --- |
|  | Preoperative | Postoperative | Preoperative | Postoperative |
| 1 | 75 | 96 | 73 | 95 |
| 2 | 72 | 94 | 70 | 92 |
| 3 | 68 | 91 | 66 | 88 |
| 4 | 65 | 89 | 63 | 86 |
| 5 | 63 | 88 | 61 | 85 |
| 6 | 60 | 86 | 58 | 83 |
| 7 | 59 | 84 | 55 | 81 |
| 8 | 60 | 87 | 53 | 84 |
| Mean ± SD | 65.3 ± 5.9 | 89.4 ± 4.1 | 62.4 ± 7.0 | 86.8 ± 4.7 |
| P value | <0.001 |  | <0.001 |  |

Note: SD = standard deviation; Paired t-test was used for the comparison of preoperative and postoperative scores.
